# A study of young peoples' attitudes to opportunistic Chlamydia testing in UK general practice

**DOI:** 10.1186/1742-4755-5-11

**Published:** 2008-12-19

**Authors:** Joanne Heritage, Melvyn Jones

**Affiliations:** 1 University College London Medical School, Department of Primary Care & Population Health, 2nd Floor, Holborn Union Building, Archway Campus, Highgate Hill, London, N19 5LW, UK

## Abstract

**Objective:**

The objective of this study was to assess young people's perceptions of being offered a chlamydia screening test in United Kingdom (UK) general practice.

**Methods:**

This is qualitative study that uses focus groups and individual interviews with young adults (age 16 – 18) to assess their views.

**Results:**

These young adults were a difficult group to gain access to. Two focus groups, one in a school, the other in a general practice (family practice), and 2 individual interviews were undertaken (total sample 18). Respondents were unfamiliar with Chlamydia, but broadly aware of sexually transmitted infections. General practice (family practice) was perceived as an acceptable place to deliver opportunistic screening, but participants felt that tests should not be initiated by GP receptionists. Novel delivery routes such as schools and "Pub"/Bar dispensing machines were discussed. Issues around stigma and confidentiality were also raised.

**Conclusion:**

Opportunistic Chlamydia screening in UK general practice (family practic seems acceptable to young adults. While this is a difficult group to gain access to for research, attempts need to made to ensure acceptability to users of this programme.

## Background

Chlamydia is a common, important and treatable sexually transmitted infection. Yet Chlamydia infection is asymptomatic in up to 70% of women and 50% of men and left untreated can result in pelvic inflammatory disease, leading to infertility and ectopic pregnancies in women [1]. The prevalence of Chlamydia is estimated to be up to 3% in young adults [2,3]. Prevalence in general practice (GP) or family practice, at the time of cervical smear testing is 2.9% [4]. Young people are particularly vulnerable to Chlamydia infection [5-7], with the under 25s having nearly twice the prevalence of the rest of the population. Chlamydial infection and its consequences cost the NHS in the United Kingdom (UK) in excess of £100 million annually. There is also an important relationship between sexual ill health, poverty and social exclusion [8].

There is evidence to support opportunistic Chlamydia testing (OCT) of sexually active women aged under 25 years, especially teenagers and this is supported by women [9] and by the UK National Strategy for Sexual health and HIV [10]. There is however, some debate about the effectiveness of the proposed model, [11] and the impact of a positive diagnosis for patients [12]. The focus for screening has traditionally been in family planning and genitourinary (GUM) clinics [13]. OCT however, will also be available in UK general practice [1] (family practice) but there are coverage, [14] logistical and commissioning issues to be resolved [1]. Targeted Chlamydia testing screening in primary care is used in other countries [15].

Primary care staff have voiced concerns regarding administering OCT in general practice (family practice). UK GPs report difficulties with screening for Chlamydia, citing time issues involved in "discussing sexual health issues, gaining consent, and performing the test procedure and to ask about contact tracing" [16]. In the pilots that have been conducted, it has often been left it to reception staff to approach patients about testing; a role they feel neither qualified in, nor comfortable with [17,16,18].

Public knowledge about Chlamydia and the consequences of untreated infection is poor, [19,20] suggesting the motivation to be tested will be low [16]. It has also been found that those with the most sexual partners, are the least likely to be registered with a General Practitioner (GP)/family physician, and consult GPs less often than those with fewer sexual partners [21]. OCT in general practice (family practice) may therefore miss those most at risk [22]. In the US, young people's beliefs about Chlamydia screening programmes revealed a lack of knowledge about Chlamydia and confidentiality concerns regarding around self testing at home [23].

The UK Department of Health (DoH) (2004) National Chlamydia Screening Programme (NCSP) aims to offer opportunistic screening to all those who are sexually active under 25 years of age, attending a variety of health care settings in England by 2008 [24]. The screening test involves self testing using urine and vulval swab methods, which is both highly sensitive, and acceptable to users [25,26].

### Research Aim

To explore the attitudes of young people towards opportunistic Chlamydia testing in general practice.

## Methods

This was a qualitative study using audio-taped focus groups (FG), and interviews with young people[27] The FG was led by a moderator and observer, with both making field notes during and after the group. If insufficient participants could be recruited, individual interviews were to be used as a "fall back" option.

### The Focus Group/Interview Process

An interview schedule or topic list was used (see appendix). The core schedule remained the same throughout the study, but was elaborated iteratively from previous sessions. The format of each FG/interview was identical. The participant/s were introduced to the characters of a similar age: Louise and Chris. The participants were encouraged to speak about the experiences of Louise and Chris rather than their own.

Participants were allowed to handle the test kits. There were two types of kit: the female only kit contained a vulva-vaginal swab and the male and female kit contained a urine pot. Both contained a DoH patient information leaflet, and instructions on how to carry out the test. The process of the FG and interviews followed guidance [28,29] on how to conduct both type of activities and how best to observe the participants. Krueger & Casey suggest, 'moderators should try to restrict head nodding' [28]. The moderator accepted all points of view without showing any preference to any particular ideas and declined any questions regarding the nature of the subject material until the end of the discussion, so that the participants drew all their ideas purely from their own knowledge and what they had managed to glean from the DOH leaflet.

### Respondent Validation

Issues raised during data collection were summarised, by the observer so participants could verify the data. The moderator and observer held a recorded debriefing after each FG, which was transcribed and verified. Key themes identified in FGs were used to inform subsequent FGs so that the data collection and the analysis, would be iterative.

### Sampling & setting

We wanted to sample young people across a wide range of socio economic and educational backgrounds. FG participants were to be recruited from the 15–18 age group. They were to be purposively sampled from pupils at secondary/senior schools in the borough of Enfield and from among patients at a GP Surgery in Enfield in the same age range. Enfield is a suburban borough on the outskirts of London, UK. The FGs were to be conducted both in schools and the practice.

A letter of introduction was sent to the 19 Heads of the secondary schools (children aged 11–18) in the borough. The schools were selected using the UK Office for Standards in Education (OFSTED) report data, with the aim of recruiting from schools above and below national average academic achievements. Volunteer participants were to be recruited following a short verbal presentation on the study.

A similar method was planned for the GP sample, with the aim of capturing the views of those, not in education. Written information about the study was provided to both groups. The participants from the practice population were recruited by means of a letter and reply slip sent by the practice. Informed consent would be obtained from participants over 16 or from the parents/guardians of participants under the age of 16.

### Ethical Approval

Ethical approval was obtained from the Barnet, Enfield & Haringey Local Research Ethics Committee.

### Ethical Issues

#### Confidentiality

Personal information may have arisen in the course of discussion, so it was necessary that the investigators and participants alike respected confidentiality.

#### Stress to the participants

The discussion was related to sexual behaviours and although personal information about the participant's sexual activity was not explored, this may have caused concern. Boundaries were discussed prior to the discussion and monitored by the observer.

#### Discussing matters related to sex with those under the age of consent

Special precautions were in place for those under 16, but were not needed. In the UK consent from children can be obtained if they are adjudged to pass the Fraser competence rules, where "children aged under 16 who have the legal capacity to consent to medical examination and treatment, providing they can demonstrate sufficient maturity and intelligence to understand and appraise the nature and implications of the proposed treatment, including the risks and alternative courses of actions" [30].

### Analysis

The audiotapes were transcribed verbatim and were validated against the audiotapes. The moderator and the observer coded the responses into categories based on the original research questions and other opinions that arose during the FG and interviews using the 'Long-Table Approach', [28,29] where each quote is categorised according to the question it answers/offers an opinion on. The coding is repeated until satisfactory concordance is reached. All the responses to each question are then summarised in a descriptive summary and major themes identified from this data.

The concepts were coded and used to link segments of narrative to create categories with common properties. Finally two researchers coded the transcripts into the emergent domains. Saturation of data was achieved when no new issues were arising in the focus groups or the interviews.

### Expected outcomes

1. Data on whether young people believe that General Practice is an appropriate setting for OCT.

2. Data on alternative settings for OCT.

3. Data on how the subject of OCT should be approached.

4. An analysis of the above qualitative data for any other major themes that emerge from the FGs.

## Results

As might be expected with the nature of the subject and the age range of the participants, the study protocol diverged from plan and we needed to use individual interviews, in addition to the planned focus groups (but we stayed within what had been agreed with the ethics committee).

### School population

Only one school (from 19) agreed to take part. JH was invited to attend the year 12 (pupils aged 16–17) assembly, but the school had inadvertently booked another speaker. Fortunately the school had advertised the study and had recruited 12 volunteers to participate (more than the requested 6 participants). Rather than turn the volunteers away, a FG was conducted with 12 participants. As they were aged 16 or 17 they were able to consent to participate themselves. The group consisted of 12 participants, 1/3^rd ^male, with varied ethnic backgrounds, including students of white, black Caribbean, south Asian and oriental backgrounds.

### Practice population

Practices do not maintain data on young people's educational status, so we were unable to target specifically those outside of full time education. We wrote to 403 of the practice patient population of 15–18 year olds; ten agreed to take part, 45 declined to participate and three letters were returned as the "addressee not known". It was only possible to contact six out of the ten practice participants who had agreed to participate despite repeated attempts to contact by letter and phone.

Some that declined gave reasons such as family or personal illness and school commitments

'apologies – studying for GCSEs* and can't really spare the time'.

(*GCSE: General certificate of secondary education- the UK high school exit exam)

Two replies written in the same handwriting were replies from patients belonging to the same family, which might imply a parent did not wish for their children to participate in the study.

The practice focus group consisted of 4 participants, age 16 to 18 with an even gender mix and from white British backgrounds.

Two "GP practice" respondents were unable to attend the focus groups and so two "one to one" interviews were conducted in their homes (with a friend or family member present). None of the participants in the study had parents present during the interview. These respondents were white British women, aged 16 and 17.

### Themes arsing from interview/focus groups

#### Attitudes to Opportunistic Testing in General Practice

In exploring young people's attitudes to OCT in General Practice, none of the participants were adverse to the idea of opportunistic Chlamydia testing (OCT).

*'I think it's a good idea to give that opportunity to young people because I don't think that age group would just decide to do it without someone approaching them first*. (White female, I2) [34]

However, participants used words such as 'embarrassed' and 'scared' to describe how the characters might feel if they were given an OCT kit.

#### General practice, an acceptable place for OCT

There appeared to be no objection to the use of General Practice as a location for screening.

*'The thing is, if you don't make it available in General Practice, people are going to have to make a special journey to, like a sexual health centre or hospital or wherever. They may think "Well I haven't got it, so there's no point in me getting a test so why make the effort to go there?" Basically it's simpler just to have it at the GP*.' (White male, FG1) [52]

There were however, some concerns about the use of General Practice as a screening location. In the FG all present were surprised that they rarely saw their peers at the doctors.

*'Young people rarely go to the doctor, I think.' *(White male, FG1) [12]

#### Female only screening

Both male and female participants were concerned about the prospect of targeting females only for opportunistic Chlamydia screening.

*'I mean, what's the point in testing just women if men are also at risk?' *(White female, FG1) [18]

Participants in both the FGs realised that the reason that females might be targeted more than males was because the Chlamydia complications were borne more heavily by females.

#### Sexual Partner Tracing

Partner tracing was naturally a cause for concern and participants were not aware that they may be asked to approach their own past sexual partners if they were to test positive for Chlamydia. Some participants raised the issue that some people do not know their sexual partners well enough to enable partner tracing.

*'what about if you had a one night stand or something like that and you actually don't know this person that well? What do you do then? *(White female, FG1) [45]

#### Concerns About the Use of Receptionists

Although the participants agreed there was a need for OCT, most expressed serious concerns about the use of receptionists as the point of OCT recruitment for several different reasons.

*'If you request it or if the doctor talks to you about it first, it's fine but if you're given it out of the blue, like going up to the desk and that and "here, have this (OCT kit)", it's a bit intimidating I think.' *(White female, I1) [46]

There were concerns about the lack of privacy in the reception area. When asked about what other patients in the waiting room might think about young people receiving testing kits, this participant responded:

*'They might feel a bit ashamed as well because...other people in the room that might see the packet being handed to them..., they'd be thinking, that person might have Chlamydia. ...then they are all having a discussion about the person's life,' *(Black female, FG2) [69]

*'Then again, would that be written across it?' *(Referring to the 'Chlamydia Screening Programme' label on the envelope) (White female, FG1) [21]

It was evident that the participants felt that OCT was a sensitive issue and required tact and privacy. The participants felt that young people would want to ask questions about OCT, that might not be appropriate to ask a receptionist or in the reception area.

*'I think it should have been the doctor (that handed out the testing kit). Just someone to explain what this is, why has it been given to me. What Chlamydia is; just more.' *(White male, FG1) [36]

All the participants thought about how a young person might feel if they were recruited to the programme at the reception desk, in front of their parents. Most of the comments demonstrated strongly negative attitudes to this situation.

*'It would be excruciatingly embarrassing.' *(White male, FG1) [8]

*'I'd feel embarrassed cos then it won't be a secret. If my parents were exposed to it as well, I would be more ashamed, then I wouldn't be able to look at their face and talk to them face to face as I used, cos I would know, that they know what I have now... especially if my Mum was with me.' *(Asian female – wearing a Hijab*, FG2) [75]

(* a headscarf worn by women from more observant Muslim traditions)

Surprisingly though, some participants felt that the presence of their parents might be a positive factor.

*'A person like Louise^α ^might actually feel okay and feel good cos she'd have the support of her Mum there. She might feel a bit nervous about taking this whole test and she (her mum) might be able to calm Louise down so she can like go and do it properly.' *(Black female, FG2) [54]

There was a strong feeling that it should be the young person's GP that should introduce the idea of Chlamydia testing and distribute OCT kits, even when they had gone in for a non-sexually related problem.

*'The doctor would have to deal with the issue that Chris^α ^actually went in for, first of all and then just say, "by the way, I can give this thing so that you can get tested if you want to."' *(White male, FG1) [43]

The participants demonstrated great sensitivity regarding the limited amount of time that a GP has with their patients.

*'But then that takes time away from treating other patients if you have to spend five minutes explaining every time an over 16-year-old comes into your surgery, it would take you quite a long time.' *(White, Female FG1) [38]

The participants were asked whether the possibility of OCT taking place at their doctor's surgery would dissuade them from visiting their GP. This question elicited a range of views.

*'I don't think it would; personally, it wouldn't affect how often I went to the doctor' *(White male, FG1) [47]

however, one participant felt that it might.

*'Some people might be scared of going to the GP if they are thinking they are gonna get this packet every time.' *(Black female, FG2) [54]

When asked how Louise would feel about talking about Chlamydia if she had known her GP from a young age or that her GP was also her parents' GP, participants demonstrated a high regard for the GP's professionalism and their rights to confidentiality.

*'If the GP was a professional, he wouldn't tell her parents anyway.' *(White female, I2) [15]

#### Other venues for OCT

Schools were suggested not only as an alternative recruitment location, but also as a method of supporting OCT carried out at other sites.

'If you educated them at school, they would know what Chlamydia is all about and by the time they go to their GP and they hand that pack out, they'd know what it's for.' (*Black African male, FG2*) [37]

A surprising suggestion as a method of providing OCT was aired.

*'Perhaps like, in pub (Bar) toilets, or wherever you have condom machines, also have a separate machine that you can get the pack from.' *(White male, FG1) [26]

But this idea was not well received by others.

*'buying tampons, it's not a problem but something like Chlamydia, it's got like, more of a stigma attached to it and so I don't think that many people would buy it *(a test kit)*in a toilet.' *(White female, I2) [96]

*'I dunno about that, a bunch of rowdy lads, a few pints, Chlamydia testing... I mean you know where this is heading.' *(White male, FG1) [28]

The idea was not completely written off by all participants with OCT kits likened to kits already widely available in the public domain.

'But if you do that, over time people gradually get used to it, like pregnancy tests now... after a while people would be more comfortable with it.' (*Black African male, FG2*) [36]

#### Urine Testing the Preferred Method

The participants were asked whether they felt Louise and Chris would be able to carry out the tests having read the instruction leaflets.

*'It's really clear like what to do, but if you're a girl and you haven't used tampons... and this is the only option that you're given, I think that you'd be too scared to do it because you wouldn't know if you were doing it right.' *(White female, FG1) [60]

Although the testing kits were introduced as the 'female only testing kit' and the 'male and female testing kit', the females very much focused on the female testing kit and did not realise that they had the opportunity to make use of either.

*'I didn't know that you could do either. I know what I would go for, I would go for the urine test, I'm sure Louise would as well.' *(White female, FG1) [31]

Most female participants were not very positive about using the female only testing kits.

*'Ouch! (Looking at the female only testing kit) I wouldn't do it.' (White female, FG2) *[15]

*'I've got good aim, its still better than bloody, (the participant motions self swabbing) I ain't putting that in there! (White female, FG2) *[44]

It was felt that young people would have no problems using the urine testing kit. With regards to the self-taken vulva-vaginal swabs, only one participant felt that she had no preference to either testing kit. Most females thought it would depend on the young person and her experience with things such as tampons as to whether she would consider and manage to take the test.

## Discussion

The results of this study would suggest that the participants accept the necessity of the NCSP and are happy for the General/Family Practice to be one venue for screening for Chlamydia. Participants felt strongly that it should be their own GP or Practice Nurse who approached them, rather than being recruited by reception staff. It is questionable if GPs' receptionists would have the time or skills to assess if a person was competent in order to recruit them to the NCSP as "The screening-initiator is responsible for ensuring that any under-16 year old being offered screening is competent to make an informed decision based on the Fraser Guidelines" [31]. According to Perkins, [21] receptionists themselves objected to only partially being involved in patient care, and found that, like our participants, 'reported 'embarrassing' instances or difficulties with patients accompanied by a parent or partner.' Other literature paints receptionists as being perceived by patients as obstructive or intrusive [32]. However full team engagement including reception staff boosts uptake rates [33].

All of the participants agreed that NCSP should target both males and females. This does not fit with the UK Government Dept of Health Information leaflet (2003), which states 'Men will not routinely be included in this screening programme".

One interesting suggestion is using schools to distribute testing kits. This is just one of many ideas for OCT to take place outside of the GUM setting that have been proposed. A press release by the DOH, 'Chlamydia screening on the high street', describes making Chlamydia screening available in pharmacies. Other initiatives include "pee in a pot" days held at military bases, colleges and youth settings [34]. Pavlin reports a desire for similar wide ranging accessibility of methods, internationally[9]. However, non invasive testing [35] from primary care[36] remain the preference in the UK and internationally [37].

### Strengths and limitations of this study

Although there are limitations to our data, the study does give us an important (although incomplete) insight into the beliefs of this group. This, unsurprisingly was a difficult study to obtain participation and stakeholder (Ethics committees, Schools, Primary Care Trusts Research & Development bodies) approval for. We did eventually recruit and manage to talk to young people from a wide-ranging population in terms of gender mix and ethnicity within the 15–18 age group, about OCT. The participants from the school were all from year 12. This means that selection bias affects the results for this age group, as they have remained in education after the minimum legal leaving age. We had mixed fortunes with regard to recruiting those who were not in full time education, in that we made initial contact but ultimately unsuccessful in doing the interviews (despite several attempts). This means that it may not necessarily be possible to generalise the views obtained in this study to the working and unemployed population in the 16–18 age group.

Is this research in a London borough generalisable to the UK? The UK prevalence of Chlamydia is broadly similar to the rest of Europe [38]. Enfield is a borough that has a broad socio economic and ethnic mix; it contains wards in both the 20% most deprived and the 20% most affluent areas in England [39] and educational attainment levels very similar to the UK. Notwithstanding this, our study school has GCSE and A Level (pre university exams in the UK) result attainments that are below the Enfield and National average [40]. In Enfield, 23% of the population belong to black and minority ethnic groups [41] and this was reflected in the ethnic make up of our respondents (including our ability to incorporate the views of a young Muslim woman). Ensuring representation of these ethnic groups is especially important, as the results from the first full year of the NCSP found that risk factors for Chlamydia "positivity" included non-white ethnicity [42].

Discussing issues related to sex with young adults is difficult due to: their own social and academic time constraints, their reluctance to discuss sexual matters with adults, and parents' reluctance to admit their children are in the transition to becoming sexually active. Embarrassment about discussing issues related to sex is not however, confined solely to young adults [43].

The qualitative nature of the study enabled the discovery of novel findings and the generation of some creative alternatives to OCT such as making testing kits available via schools and vending machines in public toilets. The vignettes of "Louise and Chris" characters were used to facilitate discussion. The use of vignettes is well established in qualitative research "to allow actions in context to be explored; to clarify people's judgements; and to provide a less personal and therefore less threatening way of exploring sensitive topics" [44]. They are particularly useful when discussing sensitive topics such as sexual health [44]. Allowing participants to handle the test kits also helped contextualise their discussions.

Despite the relatively low number of participants (and low response rate) in this study, saturation point was reached on the major topics of discussion as no new topics were being raised. The use of FG, and an interview methodology improved the reliability of the results through triangulation with each method supporting the results obtained by the other method. There were limitations to our methods; our respondent validation could only be done immediately after the groups as we were unable to individually re-contact participants. However, the speed and immediacy of the process meant that we had high levels of engagement from participants in the process.

There was a real sense that the participants were speaking honestly and in a frank manner. This feeling was supported by an incident in FG two when a member of the school staff entered the room, the room fell silent. As soon as they left the room, the conversation resumed.

#### Reflexivity

It is important to note that JH is also a medical student and there was an emphasis on developing her reflexivity in order to ensure "face validity" of this qualitative data. She also has an interest in this field of study having previously worked as sexual health peer educator in schools and Girl Guides, and this could potentially have an adverse affect on the results. These experiences may have lead to preconceptions about young people and their knowledge about sexual health matters.

### Implications for Research

Enfield is one of only 16 sites funded for the phase two roll out of the UK NCSP, but none of the participants had any direct experience of OCT. A multi-site study across several differing Primary Care Trusts, some active in the NCSP and some not, should be considered.

### Practice Implications

The results of this study support the main aim of the NCSP, especially with regard to General Practice being a screening location, but has an impact on the finer details of the local Chlamydia screening plan, which each programme area develops.

The participants of this study would appear to indicate that schools are a useful site for education and promotion of the NCSP. The participants highlighted possible problems being labelled negatively if others found out about a person taking a test and even possibly testing positive. This demonstrates the care that must be taken to ensure that strict confidentiality is maintained throughout the screening process.

When partner tracing was mentioned in the final focus group, some participants said that they knew about it because they had seen episodes of "Hollyoaks" and "Nip & Tuck" (both on UK TV). Television is an excellent medium to promote sexual health issues, especially if young people can relate to the characters facing the issues. The NCSP could exploit this method of communicating issues around OCT. None of the study participants mentioned condom use, which suggests this message as always needs reinforcing with each new group of sexually active adolescents.

## Conclusion

Young people believe General Practice to be an appropriate setting for opportunistic Chlamydia testing. Schools are perceived as an acceptable alternative venue to traditional venues for Chlamydia screening, whether this be as a site to distribute screening kits or educate young people about Chlamydia and the National Chlamydia Screening Programme. The subject of opportunistic Chlamydia testing needs however, to be approached in a sensitive manner and ensuring confidentiality.

## Competing interests

The authors declare that they have no competing interests.

## Authors' contributions

JH undertook the study MJ acted as supervisor and re wrote for publication

## Appendix

### Focus Group Questions

Setting the scene

(Alter the gender of the role on the wall to suit the focus group)

Louise/Chris goes to her/his local General Practice to see the doctor about a cold that she/he has had for a week. When she/he arrives at the receptionist's desk the receptionist hands her/him an envelope. (Hand out an envelope to each participant).

How do you think Louise/Chris feels about receiving this envelope?

Do you think that Louise/Chris understands why they have been given this envelope?

Do you think Louise/Chris should have been given this envelope by the receptionist?

Do you think someone else should give out the envelope?

Do you think it is appropriate for envelopes like this to be handed out to people like Louise/Chris in a General Practice Surgery?

Where else might it be appropriate to hand out these envelopes?

Do you think Louise/Chris will follow the instructions in the envelope and carry out the test?

Is this the best way to approach/test someone like Louise/Chris opportunistically? (An explanation of what opportunistic testing is will be given.)

Do you think someone else should give out the envelope?

Give the scenario as if the GP had given the envelope.

What if Louise/Chris have known their GP since they were little? How will they feel if their GP gives them these envelopes?

What if Louise and Chris were under 16 and they were given the envelope, how would they feel?

Do you think Louise/Chris will follow the instructions in the envelope and carry out the test?

Will Louise be more likely to take the test if she receives an envelope without a star?

What will other people in the waiting room think about Louise/Chris receiving these envelopes?

Do you think it is appropriate for envelopes like this to be handed out to people like Louise/Chris in a General Practice Surgery?

Will Louise/Chris visit the GP less if they know they might receive these envelopes when they visit?

Where else might it be appropriate to hand out these envelopes?

Schools – Nurse, request, envelopes to all, test at home.

How often should this be repeated in schools?

Would Louise/Chris make use of tests available from toiled vending machines?

How would Louise feel if only women were tested?

How would Chris feel if only women were tested?

Imagine that Louise tests positive. What might happen to her following a positive result?

How will she feel about having to contact all her ex partners?

What if Louise has lost contact or never knew her ex partners?

Is this the best way to approach/test someone like Louise/Chris opportunistically? (An explanation of what opportunistic testing is will be given.)

If Chlamydia can result in such catastrophic consequences, should people be forced to test?

## Note

^α ^Vignette character (see methods)
